# Systemic therapy for recurrent or metastatic salivary gland malignancies

**DOI:** 10.1186/s41199-016-0011-z

**Published:** 2016-09-01

**Authors:** Ashish V. Chintakuntlawar, Scott H. Okuno, Katharine A. Price

**Affiliations:** Division of Medical Oncology, Department of Oncology, Mayo Clinic, 200 First Street SW, Rochester, MN 55905 USA

**Keywords:** Salivary gland malignancy, Carcinoma, Systemic therapy, Chemotherapy, Targeted therapy, Immunotherapy, Hormone therapy, HER2 therapy

## Abstract

Salivary gland carcinomas are notoriously resistant to therapy and no standard of care exists. Due to the rarity of these malignancies, various histologies, and wide ranging clinical behavior it has been difficult to standardize systemic therapy. We have reviewed clinical prospective studies in the last 15 years with salivary gland malignancies involving cytotoxic chemotherapy and biologic agents including targeted therapies such as anti-HER-2, anti-EGFR therapies, and therapies directed at c-kit. Although the results of most trials are modest at best, there has been an increase in studies for salivary cancer in recent years and there are several promising treatment approaches in evolution. Every effort should be made to treat salivary gland malignancies under a clinical protocol and/or at a large multidisciplinary practice with clinicians experienced in treating these malignancies.

## Background

Salivary gland malignancies are morphologically and clinically varied neoplasms and account for approximately 5–7 % of head and neck cancers. Salivary gland malignancies can occur throughout the upper aerodigestive tract but the majority of tumors occur in the parotid gland and other major salivary glands [1]. The heterogeneity of salivary malignancies is underscored by the World Health Organization classification, which categorizes these tumors into 24 subtypes with varying biologic characteristics, clinical behaviors, and survival outcomes [2].

Systemic therapy for salivary gland cancers has been a longstanding problem for medical oncologists. Many malignant salivary cancers are largely cured with surgery alone but oncologists routinely encounter such subtypes as adenoid cystic carcinoma (ACC), adenocarcinoma NOS, carcinoma ex-pleomorphic adenoma, mucoepidermoid carcinoma (MEC), and salivary duct carcinoma (SDC) in the recurrent and metastatic setting. The clinical behaviors of the various types of salivary cancers range from aggressive to indolent, with heterogeneity even within any given subtype, making treatment standardization a challenge. Regardless of the type of salivary cancer, the majority of patients with metastatic salivary gland cancer will succumb to the disease, and metastatic disease remains incurable.

Surgical resection is the cornerstone of treatment for salivary gland malignancies. Radiotherapy is often employed as adjuvant therapy for tumors deemed to be at high risk of recurrence or as definitive treatment when surgical resection is not feasible. The role of chemotherapy in the definitive treatment of salivary cancer remains to be defined. The potential benefit of adding platinum chemotherapy to adjuvant radiotherapy for high-risk salivary cancer is currently under investigation in RTOG 1008 (NCT01220583), and the results of this randomized trial will hopefully inform future management. For now, the use of chemotherapy in the definitive management of salivary cancers remains up to the clinical judgment of the treating physician. The primary use of chemotherapy or systemic therapy is for patients with recurrent or metastatic disease for whom surgery or radiotherapy is not possible. Rigorous testing or standardization of systemic therapies in metastatic or recurrent salivary gland carcinomas is extremely difficult due to the rarity and heterogeneity of these tumors. In this review we discuss the current knowledge about therapeutic options for recurrent and metastatic salivary gland malignancies with a focus on cytotoxic chemotherapy and biologic agents tested in the last 15 years and a look towards future therapeutics.

## Literature search

PubMed, Medline, and Ovid databases were searched for studies related to systemic therapy for salivary gland carcinomas; lymphomas were excluded. English language publications including reviews and abstracts from January 2001 to December 2015 were considered. The time frame was restricted as there is a comprehensive review of cytotoxic chemotherapy already published in 2006 [3]. References of the studies obtained were cross-referenced for additional studies. Only phase 2–3 studies were considered. Select prospective studies were included if patients were chosen based on specified inclusion criteria and if all patients were treated with a specific regimen in a standard fashion. Select case reports/series were included only for SDC as they are particularly clinically relevant. Retrospective studies and duplicate studies published as abstracts previously were excluded.

The following sections summarize key findings of recent clinical trials for cytotoxic chemotherapy, targeted and biologic agents, and combination therapies, with a brief discussion of hormone therapy for SDC.

## Cytotoxic chemotherapy

### Combination therapy with platinum

The prior review of chemotherapy for advanced salivary malignancies by Laurie et al. in 2006 reported variable modest response rates to cytotoxic chemotherapy and concluded that there is no standard of care cytotoxic chemotherapy. The most studied chemotherapeutic regimen was the historic salivary cancer regimen cyclophosphamide, doxorubicin, and cisplatin [3]. The authors concluded that there was no clear benefit for the use of triplet therapy over single agent regimens [4, 5]. We reviewed prospective studies from 2001 to 2015 investigating cytotoxic chemotherapy for all salivary gland tumors including ACC, adenocarcinoma NOS, and MEC, with the majority of trials using chemotherapeutic combinations with either cisplatin or carboplatin (Table 1). Almost half of the studies were restricted to ACC, but the other half included other histologic subtypes albeit in smaller numbers. Four studies in particular appear to show support for the use of platinum doublet therapy; all four studies included a mixed population of patients with salivary cancer, although ACC and adenocarcinoma predominated. One study by Airoldi et al. showed improved overall response with the combination of cisplatin and vinorelbine over vinorelbine alone (44 versus 20 %, respectively), and notably is one of the only studies with randomized data [6]. In addition, the objective response rate (ORR) as well as overall survival (OS) showed a trend towards statistical significance in favor of the combination arm. Another study investigating the combination of cisplatin plus mitoxantrone showed an ORR of 14 % and a median OS of 27 months [7]. A third study by the National Cancer Institute of Canada Clinical Trials Group studied platinum and gemcitabine in patients with advanced salivary cancer with disease progression (not required for ACC patients) and reported that the ORR was 24 % and the clinical benefit rate (CBR) was 82 %. Four patients out of eight with adenocarcinoma (3 per RECIST) had a partial response and one had a complete response [8]. Recently, a large cohort of 60 patients treated with cisplatin and vinorelbine showed an ORR of 23 % with better responses seen in the first-line setting (31 % first line versus 5 % second line) [9]. Triplet therapy with platinum was reported in a small study by Ross et al. investigating the combination of cisplatin/carboplatin, epirubicin, and 5-flurouracil (5-FU) in eight patients with ACC, seven as first-line therapy [10]. Despite the addition of a third cytotoxic agent, the ORR was only 12 %, further supporting the notion that triplet cytotoxic regimens have no clinical benefit for metastatic salivary cancers. Even though the ORR was modest, the median OS was still 27 months underscoring the often indolent clinical behavior of metastatic ACC and the dual challenges of studying systemic therapy and having transient disease response translate into a meaningful survival advantage.

**Table 1:** Studies with cytotoxic chemotherapy alone or in combination with biologic agents

Author (year)	Regimen	No of patients	Histology	Progression required	ORR	CBR	Median OS (months)
Airoldi et al. [6]	Vino vs Cis + Vino	20 vs 16	Ad (9), ACC (22), MEC (1), others (4)	Yes	20 % vs 44 %	65 % vs 81 %	8.5 vs 10
Gedlicka et al. [7]	Mitoxantrone + Cis	14	NR	No	14 %	79 %	27
Gilbert et al. [11]	Paclitaxel	45	Ad (17), ACC (14), MEC (14)	No	18 %	51 %	12.5
van Herpen et al.[12]	Gemcitabine	21	ACC (21)	No	0 %	52 %	NR
Ross et al. [10]	Epirubicin + Plat + 5FU	8	ACC (8)	No	12 %	75 %	27
Laurie et al. [8]	Plat + Gemcitabine	33	Ad (8), ACC (10), MEC (4), others (11)	Yes^a^	24 %	82 %	13.8
Ghosal et al. [47]	Cis + Imatinib	28	ACC (28)	No	11 %	79 %	35
Argiris et al. [50]	Bor → Bor + Dox	24	ACC (24)	Yes	0 % and 8 %^b^	63 % and 58%^b^	21
Hitre et al. [49]	Cetuximab + Cis + 5FU	12	ACC (12)	No	42 %	92 %	24
Airoldi et al. [9]	Cis + Vino	60	Ad (15), ACC (34)	Yes	23 %	57 %	10^c^

*5FU* 5-florouracil, *ACC* adenoid cystic carcinoma; *Ad* adenocarcinoma, *Bor* bortezomib, *Cis* cisplatin, *Dox* Doxorubicin, *MEC* mucoepidermoid carcinoma, *Plat* platinum, *Vino* vinorelbine, *NR* not reported

^a^not required for adenoid cystic carcinoma

^b^response rates for Bor and Bor + Dox combination respectively

^c^median survival for first-line patients only

### Single agent cytotoxic chemotherapy

With the advent of interest in biologic and targeted therapies, single agent chemotherapy trials have become rare. In 2006, an Eastern Cooperative Oncology Group study of single agent paclitaxel showed some activity (18 %) in salivary gland carcinomas but all objective responses were seen in patients with adenocarcinoma or MEC (29 % adenocarcinoma and 21 % MEC); no objective responses were seen in patients with ACC. Despite this differential response, the OS was comparable for all subtypes, again highlighting the fact that systemic therapy has no known survival benefit for metastatic salivary cancer [11]. A second trial of single agent gemcitabine exclusively in patients with ACC was completely negative with no objective responses seen [12].

## Biologic agents

The limited utility and efficacy of cytotoxic chemotherapy have compelled the need for systematic study of alternative therapies for advanced salivary gland carcinomas. With the knowledge that salivary gland carcinomas express various potential targets such as the epidermal growth factor receptor (EGFR) [13, 14], c-kit [15], and human epidermal growth factor receptor-2 (HER-2) [16], numerous trials of biologic agents have been conducted since 2003 (Table 2).

**Table 2:** Studies with biologic agents

Author (year)	Regimen	No of patients	Histology	Progression required	ORR	CBR	Median OS (months)
Haddad et al. [18]	Trastuzumab	14	Ad (7), ACC (2), MEC (3), others (2)	No	8 %	8 %	NR
Hotte et al. [23]	Imatinib	16	ACC (16)	No	0 %	56 %	7
Pfeffer et al. [24]	Imatinib	10	ACC (10)	No	0 %	20 %	NR
Guigay et al. [25]	Imatinib	17	ACC (17)	Yes	13 %	47 %	NR
Agulnik et al. [19]	Lapatinib	39	Ad (7), ACC (20), MEC (2), others (11)	Yes	0 %	78 %	NR (ACC), 13.8 (non-ACC)
Locati et al. [20]	Cetuximab	30	ACC (23), MEC (2), others (5)	No	0 %	80 %	NR
Chau et al. [27]	Sunitinib	14	ACC (14)	Yes	0 %	85 %	18.7
Jaspers et al. [44]	Bicalutamide	10	SDC (10)	No	20 %	50 %	12
Locati et al. [29]	Sorafenib	37	ACC (19), others (18)	No	16 %	73 %	NR
Thomson et al. [28]	Sorafenib	23	ACC (23)	No	11 %	79 %	19.6
Kim et al. [34]	Everolimus	34	ACC (34)	Yes	0 %	79 %	23.7
Goncalves et al. [37]	Vorinostat	30	ACC (30)	No	3 %	87 %	NR
Hoover et al. [36]	Nelfinavir	15	ACC (15)	Yes	0 %	47 %	NR
Ho A. et al. [30]	Axitinib	33	ACC (33)	Yes	9 %	85 %	NR
Locati et al. [43]	Bicalutamide + Triptorelin	17	SDC (17)	No	65 %	88 %	44
Wong et al. [26]	Dasatinib	54	ACC (40), others (14)	Yes	2 %	50 %	14.5 (ACC), NR (non-ACC)
Jakob et al. [21]	Gefitinib	36	Ad (9), ACC (18), MEC (2), others (6)	No	0 %	59 %	25.9 (ACC); 16 (non-ACC)
Dillon et al. [32]	Dovitinib	35	ACC (35)	Yes	6 %	71 %	22.1
Ho A. et al. [35]	MK-2206	16	ACC (16)	Yes	0 %	93 %	NR
Keam et al. [31]	Dovitinib	32	ACC (32)	Yes	3 %	94 %	NR

*Ad* adenocarcinoma, *ACC* adenoid cystic carcinoma, *MEC* mucoepidermoid carcinoma, *NR* not reported

### HER-2 directed therapy

Results of HER-2 directed therapy for patients with recurrent or metastatic salivary gland cancer have been disappointing to date, with the notable exception being in patients with HER-2-positive SDC [17]. One the first studies that attempted to explore the activity of trastuzumab in an unselected population of advanced salivary gland cancers was stopped early when it was found that HER-2 positive salivary gland carcinomas are actually very rare. Fourteen patients were ultimately enrolled (7 ACC) with a single response seen in a patient with MEC [18]. Lapatinib was also studied in salivary gland carcinomas with no objective responses seen. Of note, stable disease was reported in 78 % of patients and progressive disease was required prior to trial enrollment [19].

### EGFR-directed therapy

EGFR overexpression is seen in salivary gland carcinomas [13, 14] and hence single agent cetuximab and gefitinib were studied in two negative phase 2 trials. Locati et al. enrolled 30 patients (23 ACC) with no objective responses noted with single agent cetuximab [20]. Similarly, a study by Jakob et al. in an unselected population of patients with advanced salivary malignancies reported no objective responses to gefitinib [21]. Given the complete lack of response to these agents, further investigation of single agent EGFR-targeted therapy is not warranted.

### Targeted therapy for c-kit

Overexpression of c-kit was demonstrated in salivary gland carcinomas [15, 22] prompting enthusiasm for the investigation of the c-kit-directed agents imatinib and dasatinib. Three separate studies to date have investigated the use of single agent imatinib and one study has investigated single agent dasatinib. All of the imatinib studies included only patients with c-kit-positive ACC and in total included 43 patients [23–25]. One study by Guigay et al. demonstrated a 13 % ORR to imatinib [25] but the other two trials did not show any objective responses [23, 24]. Similarly, in a large phase 2 study, a second generation c-kit inhibitor, dasatinib, demonstrated no objective responses [26].

### Multi-targeted tyrosine kinase inhibitors

Several multi-targeted tyrosine kinase inhibitors (TKI) have been investigated in advanced salivary malignancies, with the vast majority of the studies being conducted in patients with ACC. The anti-angiogenic TKIs sunitinib and sorafenib have shown overall disappointing results in patients with recurrent or metastatic salivary cancers with sorafenib having the most promise with response rates ranging from (11–22 %). No objective responses were seen with sunitinib in patients with ACC, although five of 14 patients (36 %) did have stable disease and the median OS was 18.7 months; progression within 6 months prior to study therapy was required [27]. Sorafenib has been studied in two trials – one restricted to patients with ACC and one in a mixed population, with non-ACC patients potentially deriving more benefit. Thomson et al. reported an 11 % ORR and a 19.6 month median OS in patients with ACC [28]. Similarly, Locati et al. reported an overall response rate of 16 % with differential response seen in ACC versus non-ACC patients (11 vs 22 %) [29].

Several TKIs have been studied exclusively in patients with ACC; unfortunately, ACC remains an exceptionally treatment-resistant disease. Axitinib - a small molecule inhibitor of vascular endothelial growth factor (VEGF), c-kit, and platelet derived growth factor receptor (PDGFR) - produced three partial responses (9 % ORR) in a single center trial for ACC [30]. Dovitinib -an oral tyrosine-kinase inhibitor that inhibits VEGF and fibroblast growth factor receptors (FGFR) - showed minimal activity in two trials (ORR 3 % and 6 %) and poor tolerability with grade 3/4 asthenia reported in >50 % of patients [31, 32]. Genetic analysis of tumors of patients with ACC showed that a significant number of tumors had mutations involving the FGF-PI3K-AKT pathway [33], however the AKT-inhibitor MK-2206 and the mTOR inhibitor everolimus showed no responses in ACC patients [34, 35]. Similarly, nelfinavir, a proteosome inhibitor, shown to be efficacious in AKT-inhibition, also demonstrated no objective responses in patients with ACC [36]. Even though ACC might have epigenetic dysregulation as a pathogenic mechanism [33], vorinostat -a histone deacetylase inhibitor - failed in an early trial with a reported response rate of 3 % [37].

Although the search for effective targeted therapies for patients with advanced salivary cancer has been elusive, the number of agents being tested continues to increase and recent reports of prolonged disease control in well-designed clinical trials offers some hope for optimism regarding future therapies. At the annual meeting of the American Society of Clinical Oncology (ASCO) in 2016, an unprecedented number of therapeutic trials for recurrent/metastatic salivary gland malignancies were presented. In a phase II study that required documented disease progression prior to enrollment, pazopanib – a multi-kinase inhibitor of VEGFR, PDGFR, and KIT – resulted in prolonged stable disease in both ACC adenoid cystic and non-ACC patients. Although the response rate was low (two responders – 1 ACC and one non-ACC), the trial met its primary endpoint of 6-month PFS greater than 40 % [38]. Similarly a trial of nintedanib targeting VEGFR1-3, PDGFRα/β, and FGFR1-2 had a disease control rate of 75 % and a 6-month PFS >60 %, although it was not clear if disease progression was required prior to enrollment [39]. Regorafenib, a multi-kinase inhibitor of VEGFR, FGFR, and PDGFR, resulted in prolonged stable disease (>6 months) in ACC patients who had documented progression prior to initiation of therapy [40]. Notably, the microtubular inhibitor eribulin showed a 10 % objective partial response rate in a mixed population of salivary patients, but a striking 69 % of patients had some tumor shrinkage on first tumor assessment and the disease control rate was reported at 90 % [41]. Further exploration of the use of eribulin for salivary cancers appears warranted, and the verdict is still out on the role of multi-kinase inhibitors which perhaps may play a role in select clinical situations or in combination with immunotherapy. Fusion transcripts, such as ETV6-NTRK3 [42], characterize a portion of the salivary gland malignancies and are potential targets for therapy with specific inhibitors (NCT02576431).

### Hormonal therapy

Although no prospective studies have been conducted, it is worthwhile mentioning the data for androgen deprivation in SDC as several reports have emerged in recent years and results have been promising. Retrospective data from a single institution where patients were treated uniformly with bicalutamide and triptorelin showed an impressive ORR of 65 % [43]. In contrast another retrospective study showed an ORR of 20 %. However, the latter study did not treat patients uniformly and most were not treated with a gonadotropin releasing hormone receptor agonist [44]. Recently, case reports have also demonstrated the effectiveness of second-line hormone therapy with abiraterone, a CYP17 inhibitor, after failure of first-line androgen deprivation [45, 46]. Currently, a randomized trial is underway in Europe to study the efficacy of androgen deprivation therapy in androgen receptor-positive salivary cancers (NCT01969578).

### Combination therapy with cytotoxic chemotherapy and biologics

After initial enthusiasm about the possible responses in c-kit overexpressing ACC [15, 22], cisplatin was studied in combination with imatinib. Imatinib was given as an induction regimen at 800 mg daily for 8 weeks followed by the combination of imatinib 400 mg daily with cisplatin 80 mg/m^2^ intravenously every 4 weeks. If patients had stable or responding disease, then imatinib was continued as maintenance therapy. There were only three responses but the median OS was 35 months [47]. As previously mentioned, ACC frequently overexpress EGFR [13, 14]. Hitre et al. studied a regimen similar to the EXTREME regimen [48] in patients with ACC and reported a response rate of 42 % in a cohort of 12 metastatic patients [49]. Finally, a study of the combination of bortezomib, a proteasome inhibitor, and doxorubicin showed minimal response in patients with ACC although the clinical benefit rate for the combination was reported at 58 % [50]. To date, there are no biologic therapies or combinations with chemotherapy that have a clear benefit for patients with advanced salivary cancer.

### Immunotherapy

With the current landscape of cancer therapeutics shifting towards immunotherapy, the obvious question is whether patients with salivary cancer will benefit from these agents. At the present time there is very limited preclinical data for the role of immune checkpoint inhibitors in salivary gland malignancies. A recent study showed that Programmed death Ligand-1 is differentially expressed in various histologies and is a poor prognostic marker for disease-free survival and possibly for overall survival [51]. Despite the lack of robust preclinical data, clinical trials of immune checkpoint inhibitors in salivary cancer are underway. At the 2016 ASCO meeting, preliminary results of the salivary arm of the phase 1b KEYNOTE-028 trial were presented. In the study, patients with advanced salivary cancer (73 % previously treated) received single agent pembrolizumab 10 mg/kg intravenously every 2 weeks for up to 24 months. A total of 26 patients were enrolled with mixed salivary histologies. Three PRs were seen (11.5 %), all in non-ACC patients (two adenocarcinoma, one high-grade serous carcinoma). Twelve patients (46 %) had stable disease and the 6-month PFS was 20.7 % [52]. The role of pembrolizumab monotherapy in salivary cancers continues to be investigated in the phase 2 KEYNOTE-158 basket trial (NCT02628067). Combination immunotherapy or immunotherapy in combination with other agents for salivary cancer is also being investigated in ongoing trials including ipilimumab combined with nivolumab (NCT02834013), oncolytic adenovirus in combination with pembrolizumab (NCT02576431), and histone deacetylase (HDAC) inhibitors in combination with pembrolizumab (NCT02538510). The early signal of efficacy in the KEYNOTE-028 trial opens the door for cautious optimism for immunotherapy in salivary cancers, particular non-ACC histologies.

## Discussion

Salivary gland carcinomas, especially ACC, are notoriously resistant to therapy and no standard of care exists. Platinum-based chemotherapy, if chemotherapy is given, remains the best option but falls short of being efficacious enough to be considered standard of care. Rarity of these tumors, heterogeneity in behavior, and composition of subjects in each trial make it extremely difficult to systematically study potential new therapies and to compare results across trials and over time. Although in recent years there has been greater success at conducting subtype-specific trials, at least for ACC. Of note, the number of trials published for salivary cancer has steadily climbed from 0 to 1 per year in the time period 2008–2010 to 4–6 per year in the period from 2014 to 2015, a remarkable feat given the rarity of salivary cancer, and a critical step in finding novel therapeutic approaches for this disease.

As with many solid tumors, there is often a discrepancy between preclinical data, possible therapeutic mechanisms, and the results of clinical trials. We have yet to find effective targeted therapy that is widely applicable to salivary gland carcinomas. Multiple reports had demonstrated the expression of several potential targets in salivary cancers including c-kit, EGFR, and HER-2, thus prompting a new generation of trials [14–16, 22] Despite strong scientific rationale, imatinib and dasatinib failed to demonstrate clinical benefit. One confounding factor was the heterogenous criteria for c-kit staining, and the lack of ability to correlate the degree of positive of c-kit staining with response [53]. Similarly, there is no data to determine whether the response rates are correlated to any c-kit mutations. However, c-kit mutations are not very common in salivary gland carcinoma [15]. Despite the fact that the genomic landscape of ACC is known [33], there is not a clear direction forward for salivary cancers as a whole. Individualized medicine may offer the most benefit for patients with the ability to test for driver mutations in individual patients and to tailor therapy to the patient’s tumor, from both a clinical and molecular standpoint.

Despite the overall modest results of new trials of chemotherapeutic and biologic agents, there are some notable glimmers of hope on the horizon for the treatment of advanced salivary cancers. Adenocarcinoma of the salivary gland appears to be more treatment responsive with a higher likelihood of benefit from cytotoxic chemotherapy and a preliminary signal of responsiveness to immune checkpoint inhibitors [8, 9, 11, 49]. The microtubular inhibitor eribulin deserves further evaluation given the promising disease control rate and initial tumor shrinkage that was reported in a mixed population of salivary patients; further defining which subtypes benefit the most from this agent would be helpful. The use of hormone therapy and anti-HER2 therapy for salivary duct carcinoma or AR+/HER2+ adenocarcinoma is a treatment approach that should continue to be refined and investigated. The head and neck medical oncology community will be eagerly awaiting the results of future trials of immunotherapy-based treatments that hopefully will move the practice forward and break through the therapeutic plateau that has likely been reached for cytotoxic agents; further refinement of our use of targeted agents should continue. Given the clinical rarity and nuances of each salivary cancer subtype and the changing academic landscape with a steadily increasing number of therapeutic options and clinical trials, management by an experienced head and neck medical oncologist at a tertiary referral center or academic institution is preferred if possible. Patients with advanced salivary cancers should have the opportunity to participate in salivary cancer-specific trials and/or to have access to genomic testing to open the door for appropriate phase 1 clinical trials or individualized targeted treatment with the off-label use of available agents. An experienced oncologist who sees a significant number of patients with advanced salivary cancer will be able to appreciate the clinical and biologic variability even within a certain subtype of salivary cancer, and to avoid a “once size fits all” approach which has not worked for this patient population.

**Fig. 1:**
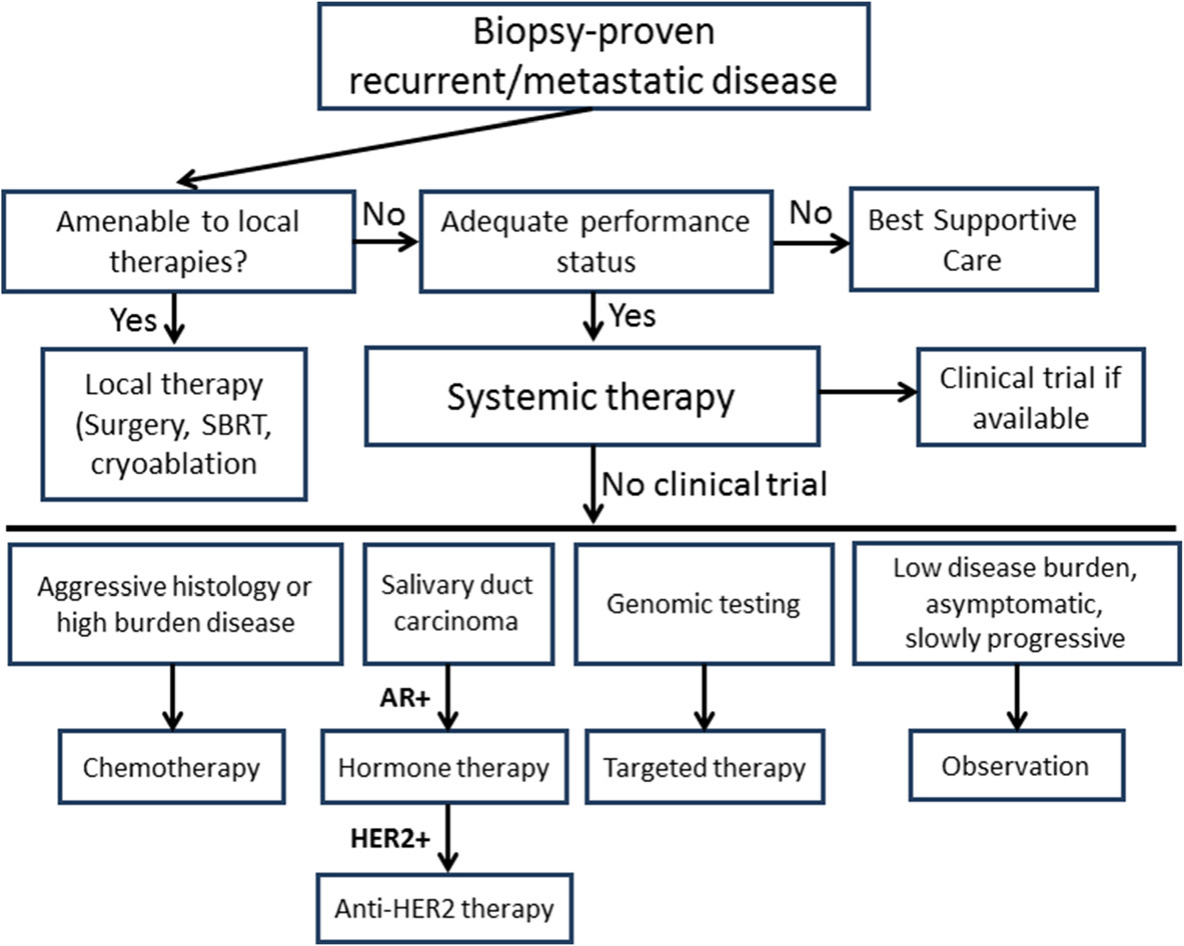
Our approach to treatment of patients with recurrent or metastatic salivary gland malignancies

From a practical standpoint, how do we typically approach our patients with advanced salivary malignancies? With the lack of high quality evidence to guide treatment and the heterogeneity of the tumors, a standard of care treatment approach to patients with recurrent or metastatic salivary is difficult to devise but with the available data we generally follow the approach outlined in Fig. 1. If the patient has a low disease burden with isolated or oligometastatic disease that is amenable to local therapies, then we typically recommend local therapy with stereotactic body radiation therapy or cryoablation. If a patient does not have an adequate performance status, then best supportive care is appropriate. In a fit patient the need to treat is balanced with the risks of systemic therapy. In a patient with slow growing, indolent disease such as classic ACC, we prefer observation. If therapy is required due to disease burden, symptoms, or an aggressive clinical course, or if the patient desires treatment (after discussion of risks and benefits), we consider systemic treatment with either standard chemotherapy, targeted therapy, or a clinical trial (preferred). In patients for whom chemotherapy is indicated we most commonly would use a platinum doublet. We routinely perform genomic testing for all patients with recurrent or metastatic salivary cancer to see if targeted therapy can be used either off-label or through a phase 1 clinical trial. For patients with adenocarcinoma and SDC we routinely perform androgen receptor staining and HER-2 testing. If they are strongly positive for androgen receptor then we prefer treating them with combined androgen–deprivation therapy with bicalutamide and leuprolide, or leuprolide alone, or with anti-HER-2 therapy if HER-2 is positive (immunohistochemistry 3+ staining). Those patients with an initial response to androgen deprivation therapy who subsequently progress will often be treated with second-line hormone therapy with abiraterone.

## Conclusion

It has been challenging to find effective therapies for salivary cancers but it is imperative that we continue to pursue research studies. Laurie and colleagues have put forth some recommendations for testing therapies in rare tumors such as salivary gland malignancies to increase coordination, avoid duplication, and increase accrual to clinical trials [54]. The studies reviewed here provide no standard of care for the treatment of salivary gland malignancies, but suggest a future landscape of heterogenous and individualized treatment for patients with salivary cancer. The advent of immunotherapy and the increase in clinical trials in recent years for salivary cancer offer hope for new therapeutic opportunities and research collaboration.

## Abbreviations

ACC: Adenoid cystic carcinoma
CBR: Clinical benefit rate
EGFR: Epidermal growth factor receptor
FGFR: Fibroblast growth factor receptor
HDAC: Histone deacetylase
HER-2: Human epidermal growth factor receptor-2
MEC: Mucoepidermoid carcinoma
ORR: Overall response rate
OS: Overall survival
PDGFR: Platelet derived growth factor receptor
PR: Partial response
SDC: Salivary duct carcinoma
TKI: Tyrosine kinase inhibitor
VEGF: Vascular endothelial growth factor
